# Exosomal FABP5 drives HCC progression via macrophage lipid metabolism and immune microenvironment remodeling

**DOI:** 10.3389/fimmu.2025.1644645

**Published:** 2025-09-16

**Authors:** Siyi Luo, Rui Tang, Ling Jiang, Qichi Luo, Junhao Fu, Bo Wu, Guowu Wang

**Affiliations:** ^1^ Department of Pathology, Suining Central Hospital, Suining, Sichuan, China; ^2^ The First Affiliated Hospital of Chongqing Medical University, Chongqing, China; ^3^ Centre for Lipid Research & Chongqing Key Laboratory of Metabolism on Lipid and Glucose, Key Laboratory of Molecular Biology for Infectious Diseases (Ministry of Education), Institute for Viral Hepatitis, Chongqing Medical University, Chongqing, China; ^4^ Department of Central Laboratory, Affiliated Jinhua Hospital, Zhejiang University School of Medicine, Jinhua, Zhejiang, China; ^5^ Department of Hepatobiliary and Pancreatic Surgery, Affiliated Jinhua Hospital, Zhejiang University School of Medicine, Jinhua, Zhejiang, China; ^6^ Department of Gynecology, Suining Central Hospital, Suining, Sichuan, China

**Keywords:** hepatocellular carcinoma, tumor-associated macrophages, exosomes, fatty acid-binding protein 5, lipid metabolism

## Abstract

**Introduction:**

The progression of hepatocellular carcinoma (HCC) is intricately linked to complex interactions within the tumor microenvironment (TME), where the reprogramming of tumor-associated macrophages (TAMs) plays a pivotal role. However, how HCC cells regulate TAM metabolism and function via extracellular vesicles, such as exosomes, remains incompletely understood.

**Methods:**

We isolated exosomes from HCC cell lines and co-cultured them with macrophages. Using proteomics, lipid analysis, flow cytometry, and animal models, we evaluated the effects of exosomal FABP5 on macrophage polarization and lipid metabolism. The role of FABP5 in tumor progression was assessed via *in vivo* experiments.

**Results:**

This study reveals that HCC cells release fatty acid-binding protein 5 (FABP5) via exosomes, transferring it to TAMs, thereby inducing significant lipid metabolism reprogramming in macrophages. Mechanistically, exosomal FABP5 promotes lipid accumulation by activating the PPARγ signaling pathway, while potentially inhibiting the PPARα signaling pathway to reduce fatty acid oxidation, ultimately driving TAM polarization towards an M2 phenotype, characterized by increased secretion of immunosuppressive cytokines and a pro-tumor phenotype. Clinical data analysis indicates that high FABP5 expression in HCC tissues correlates with poor patient prognosis. In liver-specific FABP5 knockout mouse models and HCC xenograft models, FABP5 deletion significantly suppressed tumor growth, reduced M2-type TAM infiltration and lipid accumulation, and enhanced anti-tumor immune responses.

**Conclusion:**

These findings collectively uncover exosomal FABP5 as a key mediator of metabolic and immune communication between HCC and TAMs, promoting HCC progression by remodeling the tumor immune microenvironment, and suggest FABP5 as a potential therapeutic target for HCC.

## 1 Introduction

Hepatocellular carcinoma (HCC) is the most common primary liver cancer globally, posing a significant threat to human health due to its high incidence and mortality rates (1, 2). Despite advancements in treatment, most patients are diagnosed at a late stage, leading to a poor prognosis (3). The five-year survival rate remains unsatisfactory, and the high postoperative recurrence rate is a key obstacle to long-term patient survival (4). Therefore, there is an urgent need to deeply understand the molecular mechanisms of HCC progression and identify new therapeutic targets.

The tumor microenvironment (TME) plays a pivotal role in the development and progression of HCC (5). The TME is a complex ecosystem composed of tumor cells, stromal cells, immune cells, and various non-cellular components. It actively participates in and profoundly influences nearly all biological behaviors of tumors, including proliferation, invasion, metastasis, angiogenesis, and immune evasion (6). Among the many cellular components of the TME, tumor-associated macrophages (TAMs) are one of the most abundant infiltrating immune cells (7). In HCC, TAMs typically exhibit an M2-like polarization that promotes tumor progression (8, 9). They create an immunosuppressive microenvironment by secreting inhibitory cytokines, promoting angiogenesis, and degrading the extracellular matrix, thereby directly fostering tumor cell growth and metastasis, and are associated with poor patient prognosis (9). The phenotype and function of TAMs are precisely regulated by multiple signals within the TME, and reprogramming TAMs has become a promising anti-tumor strategy (10).

Intercellular communication is crucial for shaping TME characteristics and driving tumor progression. Exosomes, a type of extracellular vehicle, are important carriers of intercellular information (11). These nano-sized membrane vesicles are secreted by almost all cell types, including tumor cells, and contain various bioactive molecules such as proteins, lipids, and nucleic acids. Tumor-derived exosomes (TDEs) can deliver their “cargo” to target cells, altering recipient cell functions and thus playing key roles in tumor proliferation, angiogenesis, invasion, metastasis, immunosuppression, and therapeutic resistance (12, 13). In HCC, exosome-mediated communication has been confirmed to participate in TME remodeling and the regulation of tumor progression (14).

Metabolic reprogramming, particularly alterations in lipid metabolism, is a hallmark of cancer (15). Tumor cells remodel lipid metabolism, including uptake, synthesis, storage, and breakdown, to meet the demands of rapid growth and adaptation to harsh microenvironments. Concurrently, the functional state of immune cells within the TME is closely coupled with their own lipid metabolic patterns. The polarization and function of TAMs are especially profoundly affected by lipid metabolism: the anti-tumor activity of M1 macrophages relies on glycolysis and *de novo* fatty acid synthesis, whereas the pro-tumor functions of M2 macrophages depend more on fatty acid oxidation (FAO) (16). The lipid-rich nature of the TME and metabolic competition between tumor cells and immune cells collectively shape the pro-tumor phenotype of TAMs.

Fatty acid binding protein 5 (FABP5) is a small intracellular lipid chaperone protein that plays an important role in fatty acid uptake, transport, and metabolic regulation (17). Its expression is significantly upregulated in various cancers, including HCC, where it is associated with malignant tumor progression and poor patient prognosis (18).Extensive research has established intracellular FABP5 as a multifunctional oncoprotein that drives tumor proliferation, invasion, and metastasis through facilitating fatty acid uptake/utilization, inducing chemoresistance pathways, and modulating cancer cell-intrinsic inflammatory signaling, among other mechanisms (19–22). Notably, FABP5 also shows an emerging critical role in immune cell regulation, participating in modulating the lipid metabolism and function of T cells and macrophages (21). Given that FABP5 is expressed in both HCC cells and TAMs and is involved in processing the abundant lipid molecules in the TME, it may play a key role in the metabolic communication between tumor and immune cells.

However, whether and how HCC cells utilize FABP5 via exosomes to specifically affect TAM lipid metabolic reprogramming, thereby shaping an immunosuppressive TME to promote HCC progression, remains an incompletely understood regulatory network. Elucidating this mechanism is crucial for a deeper understanding of HCC immune evasion and progression.

This study aims to investigate the role of HCC-derived exosomal FABP5 in regulating TAM lipid metabolism and functional polarization, and its impact on HCC progression. By focusing on the specific communication axis through which HCC cells regulate TAM lipid metabolism and immune phenotype via exosomal FABP5, this research is expected to unveil a new dimension of the complex interaction network within the tumor microenvironment. This will deepen the understanding of the mechanisms by which exosomes mediate metabolic coupling and functional remodeling between tumor and immune cells. Clarifying the role of exosomal FABP5 in HCC progression may not only offer new biomarker approaches for HCC diagnosis and prognosis but could also open new avenues for developing targeted therapeutic strategies, improving immunotherapy efficacy, and ultimately benefiting patients.

## 2 Methods and materials

### 2.1 Cell culture

Human hepatocellular carcinoma cell lines MHCC97 and Huh-7, as well as the normal human hepatocyte line THLE-2, were purchased from the American Type Culture Collection (ATCC, Manassas, VA, USA). Cells were cultured in Dulbecco’s Modified Eagle Medium (DMEM, Gibco, Cat. No. 11995065) supplemented with 10% fetal bovine serum (FBS, Gibco, Cat. No. 10082147) and 1% penicillin-streptomycin (Thermo Fisher Scientific, Cat. No. 15140122) at 37 °C in a humidified incubator with 5% CO_2_.

### 2.2 Isolation of exosomes

Exosomes were isolated from cell culture supernatants using the ExoQuick™ Exosome Precipitation Solution (System Biosciences, Cat. No. EXOQ20A-1) following the manufacturer’s instructions. Briefly, cell culture supernatants were centrifuged at 3,000 × g for 15 minutes to remove cellular debris, followed by filtration through a 0.22-μm filter (Merck Millipore, Cat. No. SLHP002SL). Exosomes were precipitated by adding ExoQuick™ solution to the filtered supernatant and incubating at 4 °C overnight. The precipitated exosomes were collected by centrifugation at 1,500 × g for 30 minutes and resuspended in sterile phosphate-buffered saline (PBS, Thermo Fisher Scientific, Cat. No. 10010023).

### 2.3 Transmission electron microscopy

Exosomes were fixed in 2% glutaraldehyde (Electron Microscopy Sciences, Cat. No. 16301) for 1 hour and then applied to copper grids coated with formvar. Samples were stained with 2% uranyl acetate (Electron Microscopy Sciences, Cat. No. 22400) and imaged using a JEOL JEM-1230 transmission electron microscope.

### 2.4 Nanoparticle tracking analysis

The size distribution and concentration of exosomes were determined using a NanoSight NS300 (Malvern Panalytical, Cat. No. NANO-NS300) with a 405-nm laser. Videos were captured in triplicate, and data were analyzed using NTA software version 3.4.

### 2.5 Western blotting

Exosome protein extracts were prepared using RIPA buffer (Thermo Fisher Scientific, Cat. No. 89900) supplemented with protease inhibitors (Roche, Cat. No. 04693132001). Protein concentration was determined using the BCA Protein Assay Kit (Thermo Fisher Scientific, Cat. No. 23225). Equal amounts of protein were separated by SDS-PAGE and transferred to PVDF membranes (Merck Millipore, Cat. No. IPVH00010). Membranes were blocked with 5% non-fat milk (Bio-Rad, Cat. No. 1706405) and incubated with primary antibodies against ALIX (Santa Cruz Biotechnology, Cat. No. sc-53727), CD63 (Santa Cruz Biotechnology, Cat. No. sc-52737), and Tsg101 (Santa Cruz Biotechnology, Cat. No. sc-365931) overnight at 4°C. After washing, membranes were incubated with secondary antibodies (Jackson ImmunoResearch, Cat. No. 111-035-003) for 1 hour at room temperature. Bands were visualized using the Pierce ECL Western Blotting Substrate (Thermo Fisher Scientific, Cat. No. 32106).

### 2.6 Macrophage culture and treatment

Raw 264.7 macrophages were purchased from ATCC and cultured in RPMI-1640 medium (Gibco, Cat. No. 11875093) supplemented with 10% FBS and 1% penicillin-streptomycin. For treatment, macrophages were incubated with exosomes (10 μg/mL) isolated from HCC cell lines and control hepatocyte lines for 24 hours.

### 2.7 Exosome uptake assay

Exosomes were labeled with PKH67 Green Fluorescent Cell Linker Kit [Sigma-Aldrich, MINI67] according to the manufacturer’s instructions. Briefly, isolated exosomes were incubated with PKH67 dye, and excess dye was removed by ultracentrifugation. Labeled exosomes were then co-incubated with recipient macrophages for 1 hour. Exosome uptake was visualized by fluorescence microscopy.

### 2.8 Flow cytometry

For flow cytometry analysis, macrophages were harvested and resuspended in PBS. Cells were then surface stained with the following antibodies for 30 minutes at 4 °C:Anti-CD163 PE (BD Biosciences, Cat. No. 561524); Anti-CD206 FITC (BD Biosciences, Cat. No. 562003). After staining, cells were washed twice with PBS and analyzed using a BD FACSAria III flow cytometer (BD Biosciences). Data was analyzed using FlowJo software (Tree Star, Inc.). Gating strategies were established to exclude debris and dead cells, and compensation was performed using single-stained controls.

### 2.9 Lipid staining

For intracellular lipid droplet visualization, cells were stained with Nile Red [Sigma-Aldrich, N3013, 1 µg/mL] or BODIPY 493/503 [Invitrogen, D3922, 1 µM]. After appropriate treatments, cells grown on coverslips were washed with PBS, fixed with 4% paraformaldehyde (PFA) for 15 minutes, and then incubated with Nile Red or BODIPY 493/503 solution for 30 minutes at room temperature in the dark. Nuclei were counterstained with DAPI [Invitrogen, D1306]. Images were captured using a fluorescent microscope.

### 2.10 Quantitative real-time PCR

Total RNA was extracted from cells using the RNAiso Plus reagent (Takara, Cat. No. 9109). cDNA was synthesized using the PrimeScript RT Reagent Kit (Takara, Cat. No. RR047A). qPCR was performed using the TB Green Premix Ex Taq II (Takara, Cat. No. RR820A) on a QuantStudio 6 Flex Real-Time PCR System (Thermo Fisher Scientific). The following primers were used:

PPARγ: Forward: 5’-GAGGAGCTGAGGAGGAGACA-3’, Reverse: 5’-GCTGCTGGTTGCTGTAGATA-3’
PPARα: Forward: 5’-GAGTGGAAAGCTGAGGAGAG-3’, Reverse: 5’-CTGCTGGTTGCTGATAGATA-3’
iNOS: Forward: 5’-GAGCTGGAAAGCTGAGGACA-3’, Reverse: 5’-GCTGCTGGTAAGGATGCTG-3’
Arg-1: Forward: 5’-GAGCTGGAAAGCTGAGGACA-3’, Reverse: 5’-GCTGCTGGTAAGGATGCTG-3’
Ym1: Forward: 5’-GAGCTGGAAAGCTGAGGACA-3’, Reverse: 5’-GCTGCTGGTAAGGATGCTG-3’
GAPDH: Forward: 5’-GAGTCAACGGATTTGGTCGT-3’, Reverse: 5’-TTGATTTTGGAGGGATCTCG-3’

### 2.11 ELISA assays

The concentrations of IL-10, IL-1β, TNF-α, and CCL17 in cell culture supernatants were measured using ELISA kits (R&D Systems) according to the manufacturer’s instructions:

IL-10: Cat. No. D1000B; IL-1β: Cat. No. D1K00; TNF-α: Cat. No. DTA00D; CCL17: Cat. No. DC170.

### 2.12 Animal models

For the *in vivo* experiments, 6- to 8-week-old male BALB/c nude mice were purchased from Charles River Laboratories. Mice were subcutaneously injected with 5 × 10^6^ MHCC97 cells (WT or FABP5-KO). Tumor volume was measured every three days using a caliper, and tumor weight was measured after sacrifice. All animal experiments were approved by the Animal Care and Use Committee of Affiliated Jinhua Hospital, Zhejiang University (Approval code: AL-JHYY202533).

### 2.13 Statistical analysis

Data are presented as the mean ± standard deviation (SD) from at least three independent experiments. Statistical analyses were performed using GraphPad Prism 8.0. Differences between groups were analyzed by Student’s t-test or one-way ANOVA. A P-value < 0.05 was considered statistically significant.

## 3 Results

### 3.1 Macrophages dominate the immune microenvironment of liver cancer and are modulated by HCC-derived exosomes

**Figure 1:**
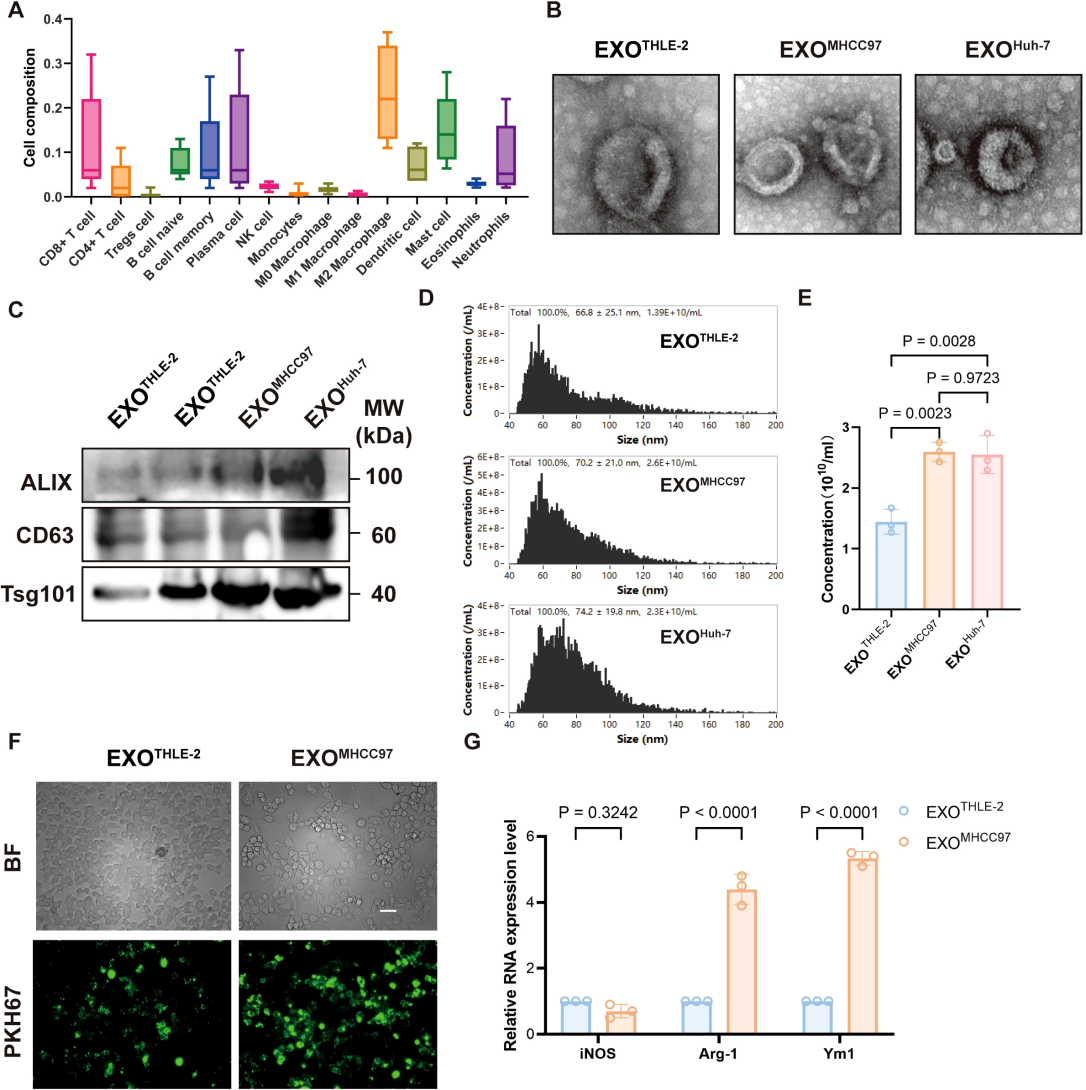
Macrophage Dominance in HCC and Modulation by HCC-Derived Exosomes. **(A)** Immunophenotyping of human HCC tissue showing relative proportions of various immune cell infiltrates, highlighting macrophages as the predominant population. **(B)** Representative Transmission Electron Microscopy (TEM) images of exosomes isolated from THLE-2 (EXOTHLE−2), MHCC97 (EXOMHCC97), and Huh-7 (EXOHuh−7) cell lines, showing typical cup-shaped morphology. Scale bar, 500 nm. **(C)** Western blot analysis of exosomal markers ALIX, CD63, and Tsg101 in lysates from EXOTHLE−2, EXOMHCC97, and EXOHuh−7. **(D)** Nanoparticle Tracking Analysis (NTA) showing the size distribution profiles of EXOTHLE−2, EXOMHCC97, and EXOHuh−7. **(E)** Quantification of exosome concentration from NTA data (particles/mL). **(F)** Representative fluorescence microscopy images showing uptake of PKH67-labeled EXOTHLE−2 or EXOMHCC97 (green) by macrophages. BF, Bright Field. Scale bar, 10 µm. **(G)** qPCR analysis of M1 marker (iNOS) and M2 markers (Arg-1, Ym1) mRNA expression in macrophages treated with EXOTHLE−2 or EXOMHCC97. Data are presented as mean ± SEM.

Analysis of Cancer Genome Atlas (TCGA) data revealed that macrophages constitute the dominant infiltrating immune cell population within the immune phenotype of human hepatocellular carcinoma (HCC) tissues, highlighting their pivotal role in shaping the liver tumor microenvironment (TME) (**Figure 1A**). To investigate the regulatory effect of liver cancer cell-derived exosomes on macrophages in the immune microenvironment, we isolated exosomes from the supernatant of liver cancer cell lines (MHCC97, Huh-7) and a control hepatocyte line (THLE-2). These exosomes exhibited typical morphology and marker profiles characteristic of authentic exosomes, as verified by transmission electron microscopy, nanoparticle tracking analysis, and western blotting (**Figures 1B–E**). Compared to exosomes from control cells, macrophages efficiently internalized these tumor-derived exosomes, evidenced by strong exosome fluorescent labeling (**Figure 1F**). Furthermore, macrophages exposed to HCC exosomes displayed a pronounced M2-like phenotype, characterized by significant upregulation of M2-associated genes (such as Arg1, Ym1) without concurrent induction of the M1 marker (such as iNOS) (**Figure 1G**). Collectively, these results underscore the dominance of macrophages in the liver cancer immune landscape and their differentiation toward an immunosuppressive phenotype induced by liver cancer cell-derived exosomes.

### 3.2 FABP5 serves as a key cargo molecule related to lipid metabolism within exosomes

**Figure 2:**
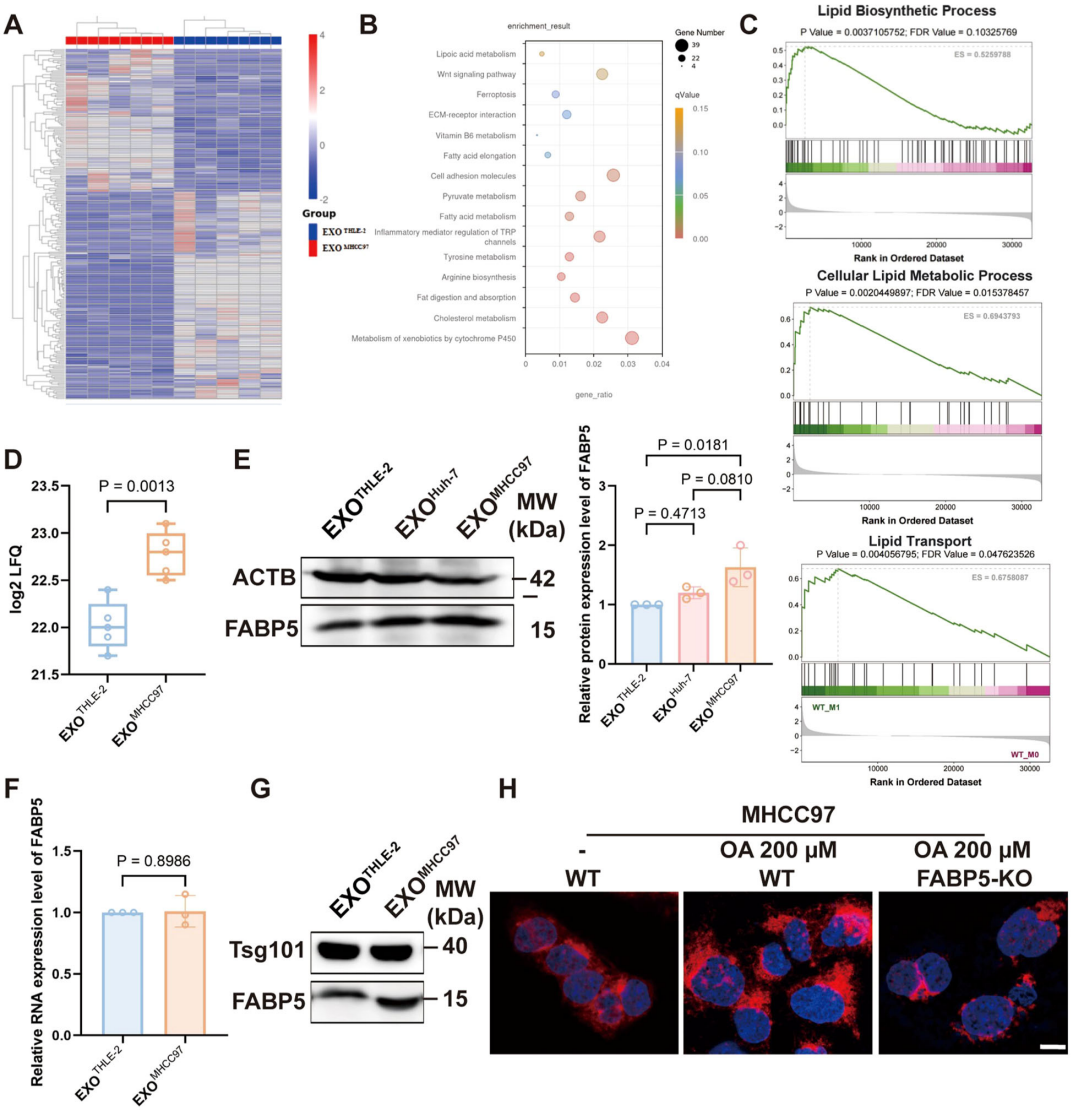
The transfer of FABP5 from HCC cells to macrophages via exosomes is associated with the regulation of lipid metabolism. **(A)** Heatmap of differentially expressed proteins identified by proteomic analysis of macrophages treated with control (EXOTHLE−2) versus HCC-derived exosomes (EXOMHCC97). **(B)** KEGG analysis of upregulated proteins in macrophages treated with HCC-derived exosomes, showing significant enrichment in lipid metabolism pathways. **(C)** Gene Set Enrichment Analysis (GSEA) plots showing enrichment of Lipid Biosynthetic Process, Cellular Lipid Metabolic Process, and Lipid Transport pathways in macrophages treated with HCC-derived exosomes. **(D)** FABP5 expression levels from the exosome proteomics data. **(E)** Western blot analysis of FABP5 in macrophages treated with EXOTHLE−2, EXOMHCC97, or EXOHuh−7. **(F)** qPCR analysis of FABP5 mRNA expression in recipient macrophages. **(G)** Western blot showing Tsg101 and FABP5 levels in exosomes derived from THLE-2 and MHCC97 cells. **(H)** Representative fluorescence microscopy images of Nile Red staining (red, lipid droplets) and DAPI (blue, nuclei) in MHCC97 wild-type (WT) cells or MHCC97 FABP5-KO cells, with or without oleic acid (OA, 200 µm treatment. Scale bar, 10 µm. Data are presented as mean ± SEM where applicable.

Proteomic profiling of macrophages exposed to hepatocellular carcinoma (HCC) derived exosomes revealed significant enrichment of lipid metabolism pathways, which was independently supported by gene set enrichment analysis showing activation of lipid biosynthesis and transport **Figures 2A-C**). Within this program, FABP5 emerged as a leading upregulated candidate in the proteomic dataset (**Figure 2D**). Western blot analysis of macrophage lysates confirmed a robust increase in FABP5 protein after treatment with HCC exosomes, exemplified by MHCC97, with quantification provided in **Figure 2E**. Importantly, FABP5 mRNA in recipient macrophages did not increase (**Figure 2F**), indicating that the rise in protein abundance is not driven by transcriptional induction. Consistent with this interpretation, direct examination of exosomal cargo demonstrated that exosomes from HCC cells contain higher levels of FABP5 than those from normal hepatocytes (**Figure 2G**). This combination of findings strongly suggests that the elevated FABP5 in macrophages results primarily from the direct transfer of FABP5 protein via HCC derived-exosomes, rather than transcriptional activation within the macrophages or donor cells. Functional validation using Nile Red staining demonstrated that FABP5 knockout significantly reduced lipid droplet accumulation within HCC cells (**Figure 2G**), suggesting a close association of FABP5 with lipid metabolic processes. Collectively, these data establish that exosomes secreted by HCC cells can transfer FABP5 protein to macrophages, a process associated with subsequent modulation of lipid metabolism pathways in these recipient cells.

### 3.3 Loss of FABP5 in HCC-derived exosomes attenuates macrophage lipid accumulation and M2 polarization

**Figure 3:**
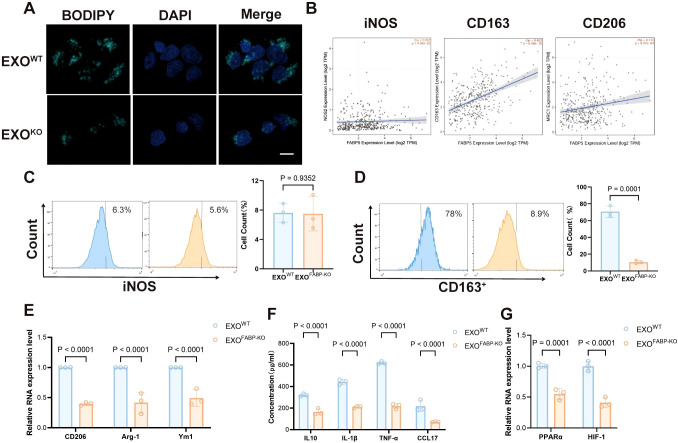
Loss of FABP5 in HCC-derived Exosomes Attenuates Macrophage Lipid Accumulation and M2 Polarization. **(A)** Representative fluorescence microscopy images of BODIPY staining (green, lipid droplets) and DAPI (blue, nuclei) in macrophages treated with exosomes from wild-type HCC cells (EXOWT) or FABP5-knockout HCC cells (EXOKO). Scale bar, 10. **(B)** Correlation analysis from TIMER2.0 database between FABP5 expression and M1 marker (iNOS) or M2 markers (CD163, CD206) in liver cancer (LIHC). **(C)** Flow cytometry analysis of iNOS expression in macrophages treated with EXOWT or EXOKO. Histograms (left) and quantification (right) are shown. **(D)** Flow cytometry analysis of CD163 expression in macrophages treated with EXOWT or EXOKO. Histograms (left) and quantification (right) are shown. **(E)** qPCR analysis of M2 markers (CD206, Arg-1, Ym1) mRNA expression in macrophages treated with EXOWT or EXOKO. **(F)** ELISA analysis of immunosuppressive cytokines (IL-10, IL-1β, TNF-α, CCL17) secreted by macrophages treated with EXOWT or EXOKO. **(G)** qPCR analysis of lipid metabolism transcription regulators (PPARα, HIF-1α) mRNA expression in macrophages treated with EXOWT or EXOKO. Data are presented as mean ± SEM.

To assess the function of FABP5 in macrophages, we treated macrophages with exosomes derived from FABP5 knockout (KO) or wild-type (WT) hepatocellular carcinoma cells. FABP5-KO exosomes significantly reduced lipid accumulation in macrophages, as evidenced by decreased lipid droplet BODIPY staining (**Figure 3A**). Analysis using the TIMER2.0 database revealed no significant correlation between FABP5 expression and the M1 macrophage marker iNOS in liver cancer but showed a significant positive correlation with the M2 markers CD163 and CD206 (**Figure 3B**). Flow cytometric analysis of macrophage markers further demonstrated that the expression of the M2 marker CD163 was significantly upregulated in macrophages treated with FABP5-KO exosomes, while the M1 marker (iNOS) was not affected (**Figures 3C, D**). qPCR results showed that the secretion of M2 markers (CD163, CD206, Arg1, Ym1) and immunosuppression-related cytokines (IL-10, IL-1β, TNF-α, CCL17) was also reduced (**Figures 3E, F**). Furthermore, the expression of key lipid metabolism transcription regulators (PPARα, HIF-1α) was also decreased in macrophages treated with FABP5-KO exosomes (**Figure 3G**). These results confirm that exosomal FABP5 promotes lipid-driven M2 polarization of macrophages, indicating that FABP5 directly participates in the reprogramming of macrophages towards an immunosuppressive phenotype in the HCC microenvironment.

### 3.4 FABP5 expression associates with poor prognosis and promotes tumor growth in liver cancer

**Figure 4:**
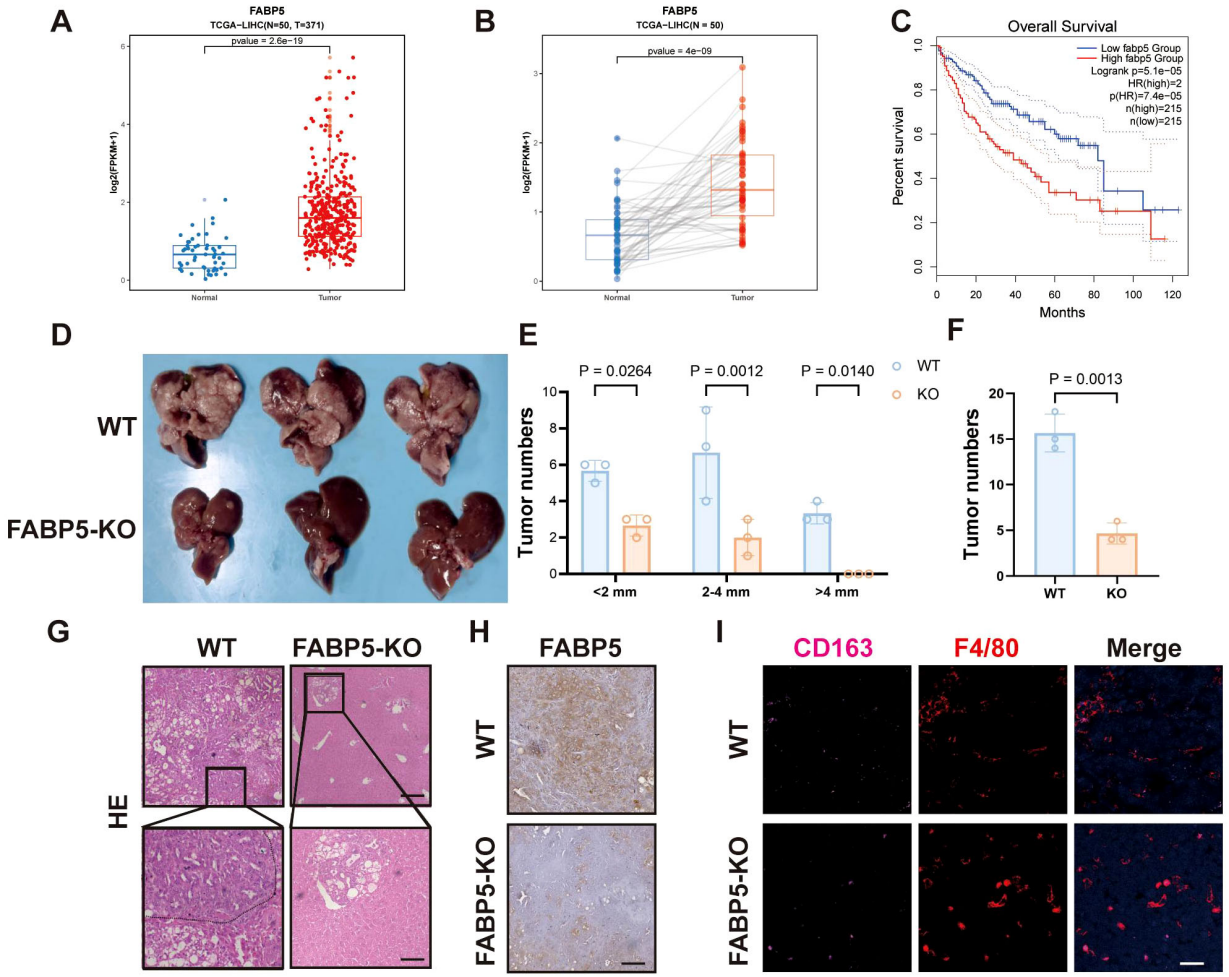
FABP5 Expression Associates with Poor Prognosis and Promotes Tumor Growth in Liver Cancer. **(A)** FABP5 mRNA expression levels in normal liver tissues (Normal) versus HCC tissues (Tumor) from the TCGA-LIHC dataset. **(B)** Paired analysis of FABP5 mRNA expression in HCC tissues and adjacent normal tissues from the TCGA-LIHC dataset. **(C)** Kaplan-Meier overall survival analysis of HCC patients from the TCGA-LIHC dataset, stratified by high versus low FABP5 expression. **(D)** Representative images of livers from wild-type (WT) mice and liver-specific FABP5 knockout (FABP5-KO) mice in a spontaneous liver tumor model. **(E)** Quantification of tumor numbers categorized by size (<2mm, 2–4 mm, >4mm) in WT and FABP5-KO mice. **(F)** Quantification of total tumor numbers in WT and FABP5-KO mice. **(G)** Representative H&E staining of liver tumor sections from WT and FABP5-KO mice. Scale bar,100 µm. **(H)** Representative immunohistochemistry (IHC) staining for FABP5 in liver tumor sections from WT and FABP5-KO mice. Scale bar, 100 µm. **(I)** Representative immunofluorescence (IF) staining for CD163 (red, M2 macrophages), F4/80 (green, macrophages), and DAPI (blue, nuclei) in liver tumor sections from WT and FABP5-KO mice. Scale bar,20 µm. Data are presented as mean ± SEM.

Analysis of clinical datasets (TCGA-LIHC) revealed significantly elevated FABP5 expression in HCC tissues compared to matched normal liver samples (**Figures 4A, B**). Survival analysis demonstrated that high FABP5 expression correlated strongly with poor clinical outcomes, highlighting FABP5 as a potential prognostic marker (**Figure 4C**). To assess the contribution of FABP5 *in vivo*, we used Cas9 mice with liver-specific FABP5 knockout achieved by tail vein injection of AAV8-FABP5-KO virus. These mice were then subjected to a spontaneous liver tumor model. Compared to the wild-type control group, FABP5 knockout mice exhibited a significant reduction in the number and size of tumors (**Figures 4D–F**). Histological examination further confirmed a decreased tumor burden and reduced infiltration of M2 macrophages (CD163 and F4/80 double-positive cells) in FABP5 knockout tumors (**Figures 4G–I**). These data establish FABP5 as a key regulator of tumor progression and immune microenvironment remodeling, with its expression directly correlated with tumor growth and macrophage polarization status.

### 3.5 FABP5 deletion alleviates immunosuppression in the tumor microenvironment and correlates with reduced tumor growth

**Figure 5:**
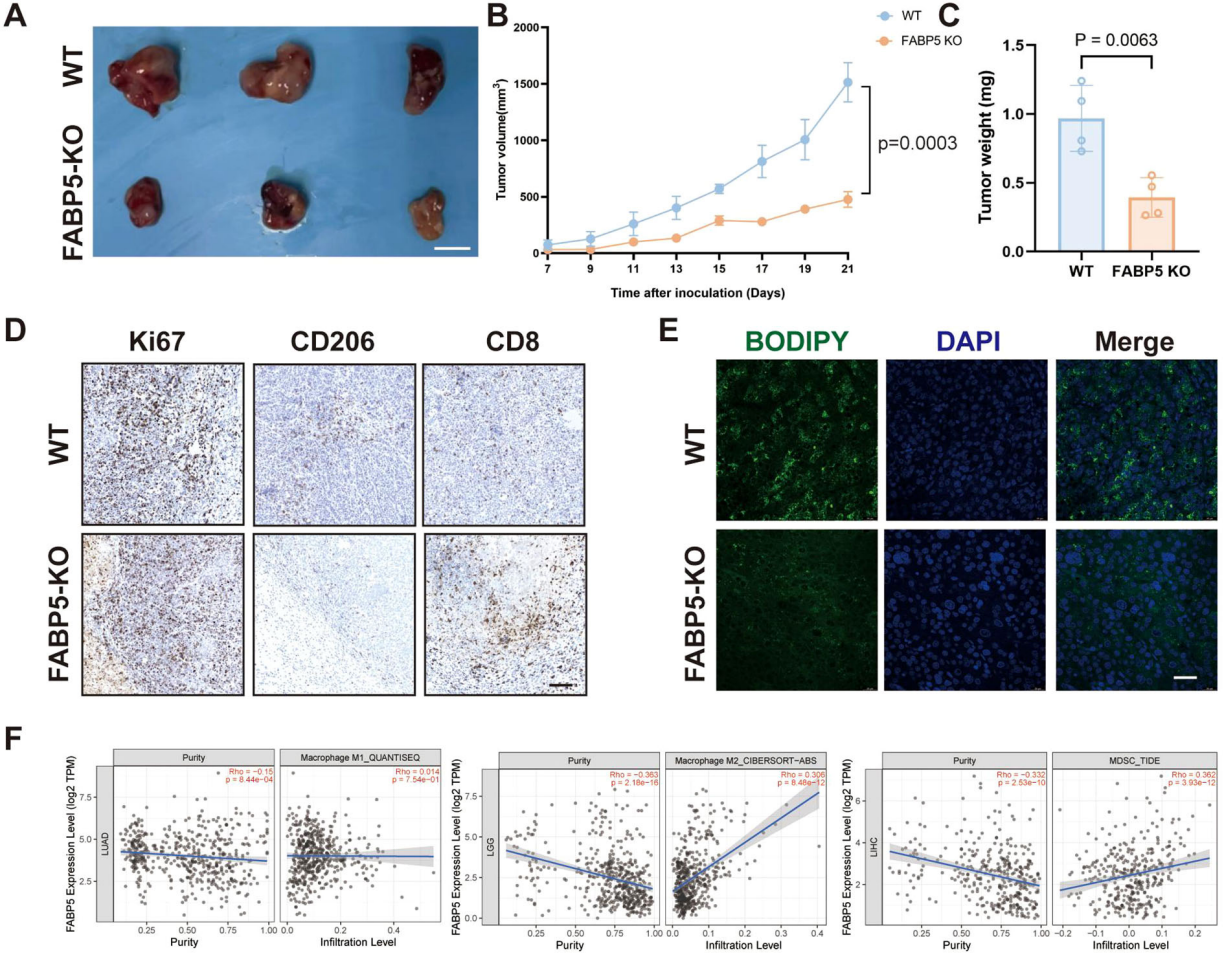
FABP5 deletion alleviates immunosuppression in the tumor microenvironment and correlates with reduced tumor growth. **(A)** Representative images of tumors from a murine xenograft model using wild-type (WT) or FABP5-KO HCC cells. Scale bar, [Specify Scale]. **(B)** Tumor growth curves of xenografts derived from WT or FABP5-KO HCC cells over time. **(C)** Tumor weights at the experimental endpoint for WT and FABP5-KO groups. **(D)** Representative IHC staining for Ki67 (proliferation marker), CD206 (M2 macrophage marker), and CD8 (cytotoxic T cell marker) in tumor sections from WT and FABP5-KO xenografts. Scale bar, 100 µm. **(E)** Representative fluorescence microscopy images of BODIPY staining (green, lipid droplets) and DAPI (blue, nuclei) in tumor sections from WT and FABP5-KO xenografts. Scale bar, 100 µm. **(F)** Correlation analysis from TIMER2.0 database between FABP5 expression and infiltration levels of M1 macrophages, M2 macrophages, and Myeloid-Derived Suppressor Cells (MDSCs) in liver cancer (LIHC). Data are presented as mean ± SEM.

In a murine tumor xenograft model, FABP5 knockout significantly impeded tumor growth, as reflected by reduced tumor volume and weight compared to WT controls (**Figures 5A–C**). Immunohistochemical analysis demonstrated substantially decreased levels of proliferative marker Ki67 and M2 macrophage marker CD206, coupled with increased infiltration of cytotoxic CD8+ T cells in FABP5-KO tumors (**Figure 5D**). Correspondingly, FABP5-KO tumors displayed diminished intratumoral lipid droplet accumulation (BODIPY staining), suggesting a reduction in lipid metabolism damage within the tumor microenvironment (**Figure 5E**). Additionally, TIMER2.0 database analysis revealed that FABP5 expression in clinical liver cancer tissues positively correlates with infiltrating immunosuppressive cells, including M2 macrophages and myeloid-derived suppressor cells (MDSCs), but not with M1 macrophages (**Figure 5F**). These findings collectively suggest that FABP5 ablation alleviates tumor-driven immunosuppression, potentially by disrupting lipid metabolism-driven recruitment and polarization of suppressive immune subsets.

### 3.6 FABP5 promotes macrophage lipid accumulation and immunosuppressive phenotype by regulating the PPARγ/PPARα pathway

**Figure 6:**
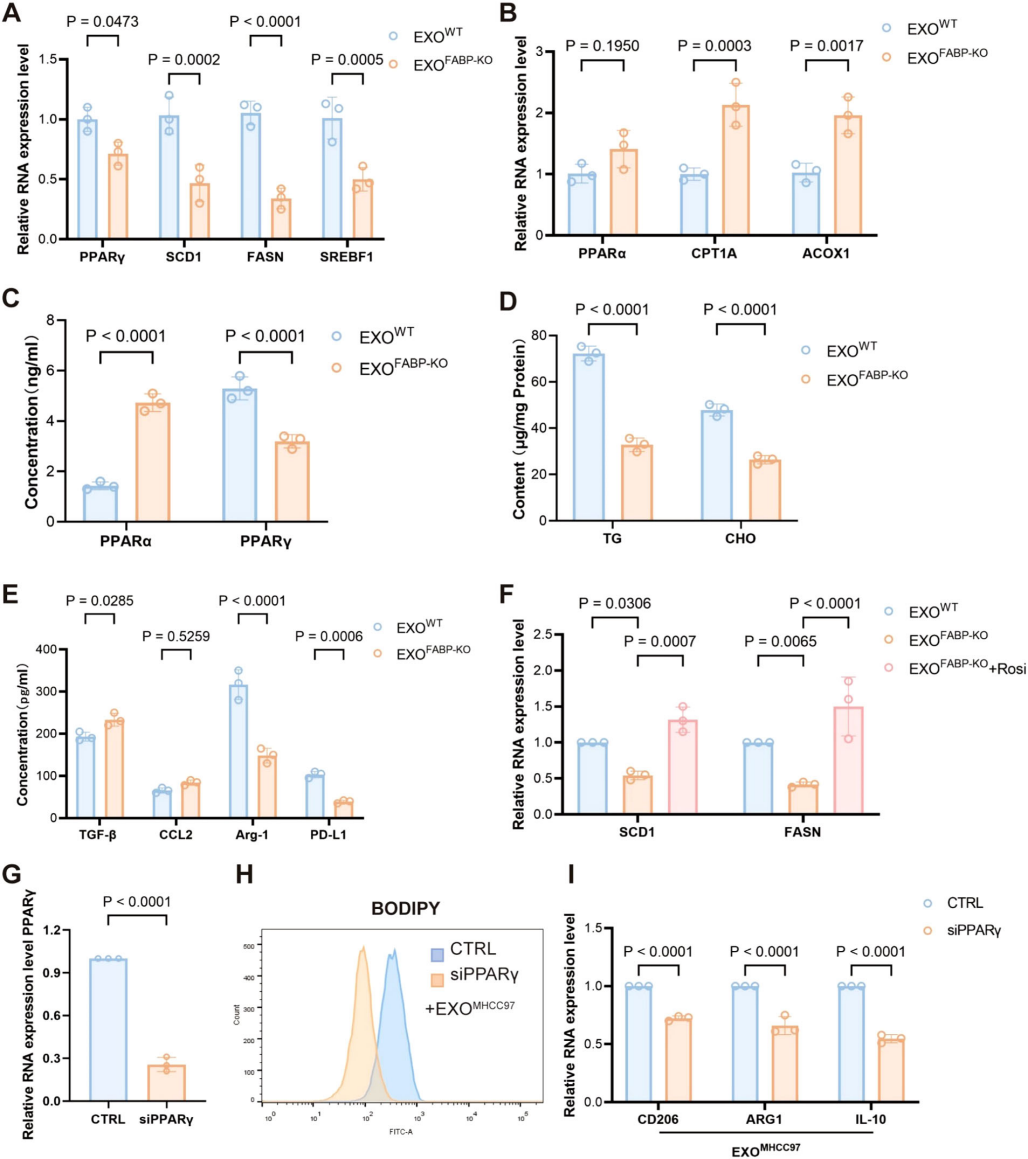
FABP5 promotes macrophage lipid accumulation and immunosuppressive phenotype by regulating the PPARγ/PPARα pathway. **(A)** qPCR analysis of PPARγ and its target genes (SCD1, FASN, SREBF1) mRNA expression in macrophages treated with exosomes from wild-type HCC cells (EXOWT or FABP5-knockout HCC cells (EXOKO). **(B)** qPCR analysis of PPARα and key fatty acid oxidation (FAO) genes (CPT1A, ACOX1) mRNA expression in macrophages treated with EXOWT or EXOKO. **(C)** ELISA analysis of PPARα and PPARγ protein levels in macrophages treated with EXOWT or EXOKO. **(D)** Measurement of intracellular triglyceride (TG) and cholesterol (CHO) content in macrophages treated with EXOWT or EXOKO. **(E)** ELISA analysis of immunosuppressive cytokines (TGF-β, CCL2, Arg-1, PD-L1) secreted by macrophages treated with EXOWT or EXOKO. **(F)** qPCR analysis of SCD1 and FASN mRNA expression in macrophages treated with EXOWT or EXOKO, and EXOKO+ Rosiglitazone (Rosi, PPARγ agonist, 5 μM for 12h) Data are presented as mean ± SEM. **(G)** Quantitative PCR analysis of PPARγ mRNA expression in macrophages transfected with control siRNA (siCTRL) or PPARγ-targeting siRNA (siPPARγ). **(H)** Flow cytometric analysis of lipid accumulation in EXO-treated macrophages transfected with siCTRL or siPPARγ, assessed by BODIPY staining. **(I)** Relative mRNA expression levels of CD206, ARG1, and IL10 in EXO-treated macrophages following transfection with siCTRL or siPPARγ, as determined by qPCR.

To elucidate the mechanistic role of FABP5 in macrophage lipid metabolism, we investigated the involvement of the PPARγ and PPARα signaling pathways. Treatment with exosomes derived from FABP5-knockout (FABP5-KO) HCC cells significantly downregulated the expression of key PPARγ target genes related to lipid uptake and storage, including SCD1, FASN, and SREBF1, suggesting that FABP5 promotes lipid accumulation via activation of the PPARγ pathway (**Figure 6A**). In contrast, FABP5-KO exosomes upregulated genes involved in fatty acid oxidation, such as CPT1A, ACOX1, and PPARα, indicating that FABP5 concurrently suppresses lipid catabolism by inhibiting PPARα activity (**Figure 6B**). ELISA analysis confirmed these transcriptional trends at the protein level: FABP5-KO exosomes increased PPARα activation while reducing PPARγ activity in macrophages (**Figure 6C**).Moreover, wild-type (WT) exosomes led to higher intracellular levels of triglycerides and cholesterol in macrophages compared to FABP5-KO exosomes (**Figure 6D**), further supporting the role of FABP5 in promoting lipid accumulation. Consistently, FABP5-KO exosome treatment resulted in significantly decreased secretion of immunosuppressive cytokines, including TGF-β, CCL2, Arg-1, and PD-L1, in the macrophage culture supernatant (**Figure 6E**). Notably, administration of a PPARγ agonist (rosiglitazone) partially rescued the phenotype caused by FABP5 deficiency, reinstating lipid accumulation and immunosuppressive cytokine expression (**Figure 6F**).

To delineate the essential role of PPARγ in macrophage reprogramming induced by exosomal FABP5, we specifically silenced PPARγ expression in macrophages using siRNA prior to treatment with exosomes derived from MHCC97 cells (EXOMHCC97). Knockdown efficiency was confirmed by a reduction in PPARγ levels of 50–80% compared to control siRNA-treated cells (**Figure 6G**; **Supplementary Figure S1A**).Upon subsequent exosomal challenge, BODIPY staining demonstrated that PPARγ depletion significantly attenuated lipid droplet accumulation compared to the control group (**Figure 6H**). Concordantly, the expression of canonical M2 polarization markers (CD206 and ARG1) as well as the immunosuppressive cytokine IL-10 was markedly downregulated following PPARγ knockdown (**Figure 6I**). Notably, PPARγ silencing alone in the absence of exosomal stimulation did not significantly elevate the expression of M2 markers (**Supplementary Figure S1A**). This result indicates that PPARγ ablation per se is insufficient to initiate M2 polarization under baseline conditions, underscore that the enhanced M2 polarization observed is primarily attributable to exosomal FABP5 rather than a nonspecific consequence of PPARγ modulation.

## 4 Discussion

The complex pathogenesis of hepatocellular carcinoma (HCC) is closely related to the dynamic evolution of the tumor microenvironment (TME), in which the reprogramming of immune cells is particularly critical. This study focuses on how HCC cells utilize exosomes, an intercellular communication carrier, and fatty acid-binding protein 5 (FABP5) to precisely regulate the metabolic state and functional phenotype of tumor-associated macrophages (TAMs), thereby shaping a microenvironment conducive to tumor growth and immune evasion (23, 24). Our research, for the first time, systematically reveals that HCC-derived exosomal FABP5 can be effectively taken up by macrophages, significantly upregulating FABP5 protein levels within these cells. This, in turn, triggers a series of lipid metabolism reprogramming events, ultimately manifesting as a substantial accumulation of intracellular lipid droplets and the polarization of macrophages towards an M2 phenotype (25, 26). This discovery provides a new molecular perspective for understanding the complex regulatory network of HCC progression and highlights the importance of exosome-mediated metabolic communication between tumors and immune cells.

In-depth mechanistic exploration shows that exosomal FABP5 regulates macrophage lipid metabolism via a dual-action mode. On one hand, FABP5 activates the peroxisome proliferator-activated receptor γ (PPARγ) signaling pathway, promoting the expression of key genes involved in fatty acid uptake, synthesis, and storage (such as SCD1, FASN, SREBF1), leading to lipid accumulation within macrophages (27, 28). On the other hand, FABP5 appears to inhibit the PPARα signaling pathway, downregulating genes associated with fatty acid oxidation (such as CPT1A, ACOX1), thereby reducing lipid β-oxidation (29). This metabolic shift of “promoting storage and inhibiting consumption” collectively drives the transformation of macrophages towards an M2 phenotype, causing them to secrete more immunosuppressive cytokines (such as IL-10, TGF-β, CCL2, PD-L1) and pro-tumor growth factors (such as Arg-1), thus creating an immunosuppressive tumor microenvironment. Clinical data analysis further confirms the key role of FABP5 in HCC, where its high expression in tumor tissues is significantly correlated with poor patient prognosis. More importantly, in both the constructed liver-specific FABP5 knockout spontaneous liver cancer mouse model and the HCC xenograft tumor model, FABP5 deletion effectively inhibited tumor growth, reduced M2-type TAM infiltration and lipid accumulation, and promoted the recruitment of anti-tumor immune cells such as CD8+ T cells. These results strongly demonstrate the central regulatory role of FABP5 in HCC progression and immune microenvironment remodeling.

Our findings are consistent with the broad understanding of the role of tumor-associated macrophages (TAMs) in tumor progression, where M2-type TAMs promote tumor growth, angiogenesis, and immunosuppression through various mechanisms (30). Exosomes, as key mediators of intercellular communication, are increasingly recognized for their role in tumor-immune cell interactions (31, 32). This study identifies FABP5 as a critical pro-M2 polarization “cargo” in HCC-derived exosomes, not only enriching our understanding of the complexity of exosomal contents but also providing new molecular targets for understanding how tumors systematically “educate” immune cells to suit their growth needs. Unlike previous studies primarily focusing on the endogenous functions of FABP5 within tumor cells, this research innovatively reveals that tumor cells can “export” FABP5 via exosomes to directly affect the metabolism and function of immune cells in the TME. This suggests that FABP5 may act as an important autocrine and paracrine signaling molecule, exerting regulatory effects beyond cell autonomy in the TME. Lipid metabolism reprogramming is a common feature of tumor and immune cells (33), and the functional state of TAMs is tightly coupled with their lipid metabolic patterns. While traditional views suggest M2-type macrophages rely more on fatty acid oxidation (FAO) (34), this study observed that exosomal FABP5-induced M2 polarization is accompanied by downregulation of FAO-related genes and increased lipid storage. This indicates that in the HCC microenvironment, the metabolic regulation of M2-type TAMs may be more complex, and accumulated lipids might serve not only as energy reserves but also as signaling molecules or substrates that directly promote M2-related gene expression and immunosuppressive functions through pathways like PPARγ activation. As a lipid chaperone protein, FABP5 may precisely regulate the transport and allocation of specific fatty acids, thereby finely tuning the activity balance of PPARγ and PPARα, a mechanism consistent with the central regulatory role of the PPAR family receptors in lipid metabolism and inflammatory responses.

Despite the robust evidence for tumor-derived FABP5-mediated macrophage reprogramming, it is essential to recognize that immune modulation in tumors is inherently multifactorial. The TME constitutes a dynamic and complex network of tumor-intrinsic signals and host-derived components—including cytokines, growth factors, stromal cues, and metabolic gradients—all of which can converge to influence the phenotype and function of tumor-associated macrophages (TAMs). Among these, interleukin-4 (IL-4) is a well-established immunoregulatory cytokine that promotes M2 macrophage polarization and has been reported to transcriptionally upregulate FABP5 expression in macrophages (35). Importantly, we employed a xenograft model using wild-type and FABP5-knockout HCC cells implanted into immunocompetent hosts under identical cytokine and immune conditions. By keeping the host microenvironment constant between groups, we ensured that differences in tumor progression, M2-type macrophage infiltration (CD206^+^), and CD8^+^ T cell recruitment were attributable to tumor-intrinsic FABP5 expression and secretion, rather than to variations in systemic cytokine levels or immune tone. These findings strongly support the conclusion that exosomal FABP5, derived from tumor cells, plays a distinct and non-redundant role in shaping the local immune landscape. While we do not exclude the possibility that host cytokines such as IL-4 synergize with FABP5 signaling, our data highlight the sufficiency of tumor-derived FABP5 to remodel the immune microenvironment, even in the presence of competing endogenous signals.

Although this study has made significant progress, some aspects require further exploration. Firstly, the universality of the findings needs to be validated in a broader range of HCC cell lines, primary patient-derived cells, and exosomes. Secondly, the specific molecular mechanisms by which FABP5 regulates PPARγ/PPARα activity, and which specific downstream lipid molecules or metabolites directly mediate the functional changes in macrophages, still need in-depth analysis using techniques such as lipidomics. Furthermore, the long-term effects of targeting FABP5 or its mediated pathways on normal physiological functions, especially liver homeostasis and systemic immune status, are critical issues to be carefully evaluated before clinical translation. Lastly, the detailed mechanisms of exosomal FABP5 entry into macrophages, including its recognition receptors, endocytic pathways, and the subcellular localization and interacting protein network of FABP5 within macrophages, are also directions worthy of future investigation.

This study systematically elucidates a novel mechanism by which HCC cells regulate TAM lipid metabolism and immunosuppressive phenotype via exosomal FABP5, not only deepening the understanding of HCC immune evasion and progression mechanisms but also providing an important theoretical basis and potential targets for developing new HCC therapeutic strategies. FABP5 itself and its levels in exosomes hold promise as candidate biomarkers for HCC diagnosis, prognosis assessment, and prediction of treatment response. More importantly, given that the immunosuppressive phenotype of TAMs is a key bottleneck affecting the efficacy of current immunotherapies (such as immune checkpoint inhibitors), targeting FABP5 or its mediated lipid metabolism pathways may reverse the pro-tumor function of TAMs, transforming “cold” tumors into “hot” tumors, thereby enhancing the sensitivity and efficacy of immunotherapy. Therefore, developing specific FABP5 inhibitors, or interfering with the release, transport of exosomal FABP5, or its interaction with TAMs, combined with existing immunotherapies, may offer a synergistic and more effective therapeutic approach for HCC patients, ultimately improving their clinical outcomes.

## 5 Conclusion

This study elucidates a critical role for HCC-derived exosomal FABP5 in reprogramming macrophage lipid metabolism to drive immunosuppressive polarization and tumor progression. Through integrated proteomic, functional, and clinical analyses, we demonstrate that FABP5-enriched exosomes are internalized by macrophages, triggering lipid droplet accumulation via PPARγ-mediated activation of lipid storage pathways and suppression of PPARα-driven fatty acid oxidation. This metabolic shift promotes an M2-like phenotype characterized by elevated immunosuppressive cytokine secretion (e.g., IL-10, TGF-β) and reduced anti-tumor immunity, as evidenced by decreased cytotoxic CD8+ T-cell infiltration. Clinically, high FABP5 expression correlates with poor prognosis in HCC patients and increased intratumoral M2 macrophage abundance. Genetic ablation of FABP5 disrupts these processes, attenuating tumor growth and immunosuppression in preclinical models. These findings position FABP5 as a central mediator of metabolic-immune crosstalk in HCC, offering a promising therapeutic target to counteract tumor-associated immunosuppression by modulating lipid metabolism in macrophages.

## Data Availability

The original contributions presented in the study are publicly available. This data can be found here: http://www.ebi.ac.uk/pride with accession number: PXD068213.

## Glossary

AAV8: Adeno-Associated Virus serotype 8
ACOX1: Acyl-CoA Oxidase 1
Arg1: Arginase 1
BODIPY: Boron-Dipyrromethene
CCL17: C-C Motif Chemokine Ligand 17
CCL2: C-C Motif Chemokine Ligand 2
CPT1A: Carnitine Palmitoyltransferase 1A
ELISA: Enzyme-Linked Immunosorbent Assay
FABP5: Fatty Acid Binding Protein 5
FASN: Fatty Acid Synthase
GSEA: Gene Set Enrichment Analysis
HCC: Hepatocellular Carcinoma
HIF-1α: Hypoxia-Inducible Factor 1-alpha
iNOS: Inducible Nitric Oxide Synthase
IL-10: Interleukin-10
IL-1β: Interleukin-1 Beta
MDSCs: Myeloid-Derived Suppressor Cells
NTA: Nanoparticle Tracking Analysis
PD-L1: Programmed Death-Ligand 1
SCD1: Stearoyl-CoA Desaturase 1
SREBF1: Sterol Regulatory Element-Binding Transcription Factor 1
TCGA-LIHC: The Cancer Genome Atlas–Liver Hepatocellular Carcinoma
