# Supplementary material for: Exosomal FABP5 drives HCC progression via macrophage lipid metabolism and immune microenvironment remodeling
Source: Front Immunol. 2025 Sep 16;16:1644645. doi: 10.3389/fimmu.2025.1644645 (PMC12479467; doi:10.3389/fimmu.2025.1644645)
Supplement: Supplementary file 1 [file DataSheet1.docx]

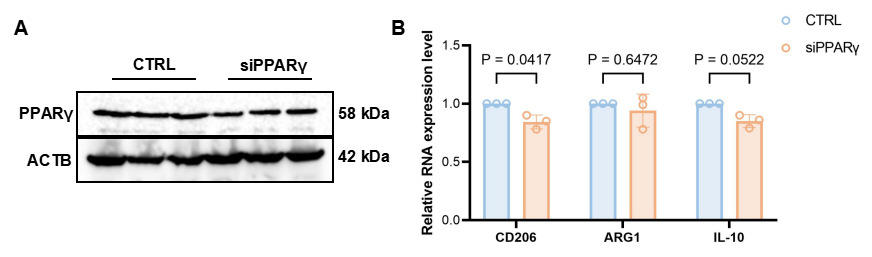


**Figure S1.** **siPPARγ lowers basal M2-marker expression in macrophages. (A)** Representative western blot analysis of PPARγ protein expression in macrophage lysates 48 hours after transfection with control siRNA (siCTRL) or PPARγ-targeting siRNA (siPPARγ), n=3. (**B)** mRNA expression levels of the M2 markers CD206, ARG1, and IL-10 in macrophages transfected with siCTRL or siPPARγ, as determined by qRT-PCR. Data are presented as mean ± SD from three independent experiments. Statistical significance was determined by t-test.
